# Visual Clutter Causes High-Magnitude Errors

**DOI:** 10.1371/journal.pbio.0040056

**Published:** 2006-02-28

**Authors:** Stefano Baldassi, Nicola Megna, David C Burr

**Affiliations:** **1**Dipartimento di Psicologia, Università di Firenze, Firenze, Italy; **2**Istituto di Neuroscienze del Consiglio Nazionale delle Ricerche, Pisa, Italy; McMaster UniversityCanada

## Abstract

Perceptual decisions are often made in cluttered environments, where a target may be confounded with competing “distractor” stimuli. Although many studies and theoretical treatments have highlighted the effect of distractors on performance, it remains unclear how they affect the*quality* of perceptual decisions. Here we show that perceptual clutter leads not only to an increase in judgment errors, but also to an increase in perceived signal strength and decision confidence on erroneous trials. Observers reported simultaneously the direction and magnitude of the tilt of a target grating presented either alone, or together with vertical distractor stimuli. When presented in isolation, observers perceived isolated targets as only slightly tilted on error trials, and had little confidence in their decision. When the target was embedded in distractors, however, they perceived it to be strongly tilted on error trials, and had high confidence of their (erroneous) decisions. The results are well explained by assuming that the observers' internal representation of stimulus orientation arises from a nonlinear combination of the outputs of independent noise-perturbed front-end detectors. The implication that erroneous perceptual decisions in cluttered environments are made with high confidence has many potential practical consequences, and may be extendable to decision-making in general.

## Abstract 2

Visual targets imbedded in distractors lead not only to judgment errors, but also to higher confidence about erroneous decisions. The signed max model provides a plausible framework for explaining the disparity between perception and reality.

## Introduction

Life is full of decisions. Perceptual decisions are usually studied in the laboratory by requiring observers to discriminate in a forced choice between two simple alternatives, judging whether a target was presented on a particular trial, or making a binary decision about some attribute of the target, such as its location, motion, or tilt. When the signal strength approaches threshold, observers become less and less sure of their responses, and to a large extent, guess. In general, the confidence with which they guess correlates well with stimulus strength as well as with actual performance [1,2].

While laboratory experiments are usually devised to simplify conditions, the psychophysical paradigm of visual search specifically investigates the ability of human observers to make perceptual decisions in cluttered visual environments, where a visual target is displayed together with a variable number of distractors. Under a broad range of conditions, increasing the number of distracting elements degrades both the accuracy and reaction time of performance [3,4]. Whereas the effects of set size have often been thought to implicate serial processing [5], many recent studies [6–12] account for search results within the framework of signal detection theory (SDT) [13] by the effects of stimulus uncertainty: the uncertainty about which stimulus is the target means that all stimuli need to be monitored, and each stimulus monitored brings with it more sampling noise, limiting performance (for review and tutorial, see [3]).

Visual search has been studied with a wide variety of tasks, including discrimination of basic features like orientation or length, letter recognition, and more complex “conjunction” tasks involving the combination of features. In this and previous studies [9,14] we chose to measure orientation discrimination, a task that is well described psychophysically [15] and based on known physiologic mechanisms [16]. Observers are briefly presented with a circular array of grating patches such as those illustrated inFigure 1, all vertical except for the target, and asked to identify the direction of target tilt (without necessarily knowing which of the targets was tilted). Performance thresholds in this task depend strictly on the number of elements in the display set, increasing with the square root of set size over a wide range (seeFigure 2 in [14]).

**Figure 1:**
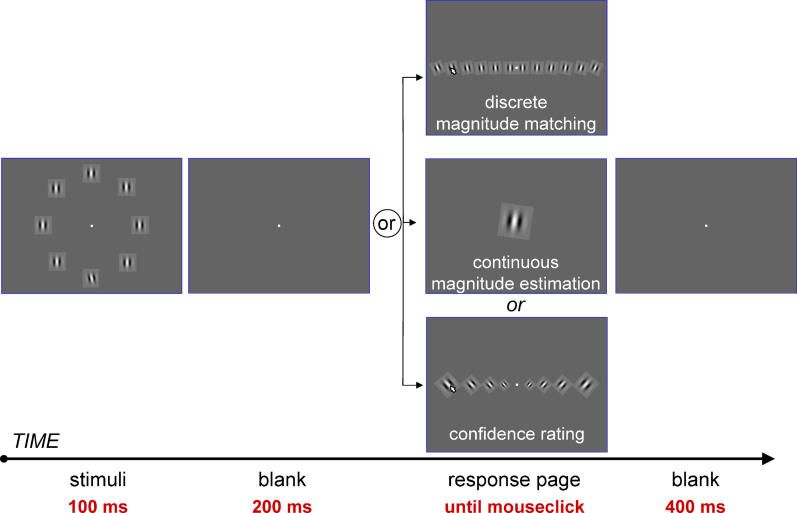
Illustration of the Experimental Sequence The leftmost panel shows a typical stimulus set (in this case a counterclockwise tilted target with seven vertical distractors) displayed for 100 ms. A blank page followed for 200 ms. Then, the response page was shown until the subject responded. In the discrete magnitude-matching task (top) we used icons representing the stimulus set (i.e., all possible orientations for the target: ± 0.5°, 1°, 2°, 4°, 8°, and 16°). Observers clicked the icon that best matched their impressions for that trial. In the continuous magnitude estimation task, a response probe resembling the target (but two times larger) appeared and could be rotated through ± 32° by lateral motion of the mouse. In the confidence rating task the icons were all ± 45° off vertical, and varied in size (from 0.5 to two times the actual stimulus size), where size represented observer confidence. After the mouse click a blank page appeared for 400 ms before the next trial. Responses were classified as correct or incorrect (depending on the chosen sign of tilt), and stored together with its magnitude match or the confidence rating.

**Figure 2:**
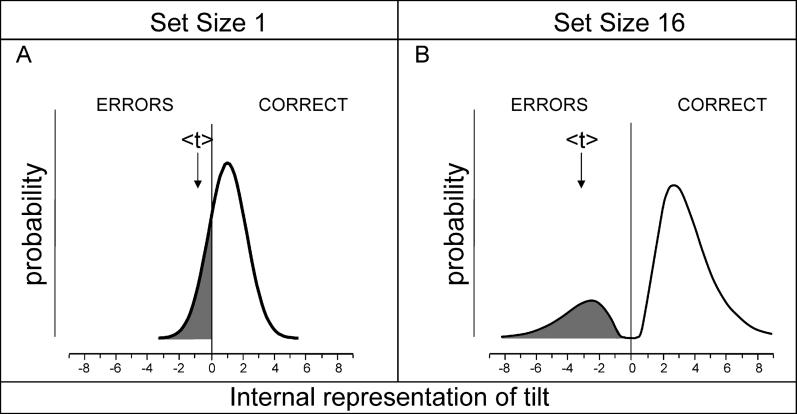
Probability Density Functions of Theoretical Internal Neural Representations of Target Tilt Pdfs are shown for when the target is presented alone (A) and together with 15 distractors (B), at target tilts that support 76% correct responses (d′ = 1). The predictions are derived from SDT, assuming a nonlinear combination rule of the output of local orientation detectors (see text).

Figure 2 illustrates a model based on SDT that predicts this result. This model assumes that each stimulus will be analyzed locally by detectors perturbed by uncorrelated neural noise. When the target is presented in isolation, the internal representation of tilt can be described by a probability density function (pdf) well approximated by a Gaussian distribution centered at the physical angle of tilt with a standard deviation equal to the presumed neural noise (Figure 2A). When the angle of tilt is equal to the standard deviation of the noise, responses will be 76% correct, the usual definition of threshold (detectability index d′ = 1). When distractors are introduced, the situation becomes more complex as observers do not know a priori which stimulus to monitor. Each stimulus should generate a noisy neural representation that can be described by pdfs like that ofFigure 2A, but centered at vertical for the distractors. If we assume that the visual system chooses the most tilted of these noisy signals (“signed max rule” [9,12]), then the internal representation of tilt at each trial will be sampled from the bimodal pdf of maxima described inFigure 2B. As the number of noisy distractor signals increases, the probability that at least one is stronger than the target increases. In order to compensate for this interference and maintain 76% correct responses, the tilt must be increased, accounting for the strong increase in thresholds in cluttered conditions.

The approach illustrated inFigure 2 leads to another strong and unexpected prediction. Not only should discriminability thresholds increase, but “observers should make more high-confidence errors when there are many distractors than when there are few” [12]. This prediction follows from inspection of the curves ofFigure 2. For set size 1, the pdf is unimodal, so most errors (shaded region) occur when the internal representation of tilt is near zero, much less than threshold resolution. Observers should consequently have low confidence in their judgment. However, for set size 16 the pdf is bimodal, so errors tend to correspond to internal representations of tilts that are distinctly nonzero, both on correct and error trials. Observers should consequently have much higher confidence in their judgment under this condition. The expected value of the perceived tilt in the error trials (t, indicated by the arrows) will be 3.2 threshold units, compared with 0.8 threshold units for the isolated target.

In this study, we present a novel psychophysical technique to probe the internal representation of orientation during visual search, combining magnitude estimation and confidence ratings with two-alternative forced-choice decisions. The results of this task confirm the prediction illustrated inFigure 2. Perceived tilt was far greater for errors made in the cluttered environment than for isolated targets. Observer confidence ratings followed a similar pattern. Although this illustration is for the basic stimulus attribute of orientation, it should clearly be generalizable to other attributes and probably also to more complex situations.

## Results

Observers were required to report both the direction and the magnitude of tilt of a grating patch, briefly presented either on its own or within a circular array of vertical distractors (seeFigure 1 andMaterials and Methods section for details). Target tilt was selected at random from 12 one-octave spaced orientations, ranging between ± 24°. In the magnitude-matching task (used for most experiments), observers indicated the direction and magnitude of the perceived tilt either by choosing a stimulus from a response set that matched the apparent tilt of the target; in magnitude estimation, observers indicated direction and magnitude by setting by mouse the orientation of a line. In the latter case, any orientation between ± 32° (including vertical) was permitted, but the direction of tilt was always defined (clockwise or counterclockwise). In the confidence-rating task, observers indicated the direction of tilt and the confidence of their decision by clicking icons of the appropriate size.

Responses were scored correct if the sign of the tilt was correctly identified, regardless of the magnitude match or confidence rating. For each subject and condition, the responses were binned into three classes of discriminability: near-threshold (67%–83% correct responses: 0.62 < d′ < 1.35), subthreshold (less than 67% correct), and suprathreshold (more than 83% correct).

### Magnitude Matches and Estimations

Figure 3 reports the results of two observers for the magnitude-matching task, and one for the magnitude estimation task, for near-threshold stimuli. The upper curves show results for set size 1, with black circles referring to normal presentation conditions and red triangles referring to conditions where the actual orientation of the target was perturbed randomly [9]. Both sets of data follow unimodal distributions, well described by a Gaussian of mean and standard deviation equal to threshold (defined as target tilt for d′ = 1) as predicted by basic SDT [13] (seeFigure 2A). External noise, in the form of random perturbation of each of the 12 possible tilt values, increases threshold (hence, mean and standard deviation), but the curve remains unimodal. However, as predicted (Figure 2B), the pattern of results for large set sizes was quite different. The data are no longer well fit by a unimodal distribution, but have two distinct peaks in perceived magnitude: one positive, corresponding to the actual stimulus; and the other negative, reflecting an illusory perceived tilt. The bimodality becomes more marked and the separation of the peaks increases as more distractors are added.

**Figure 3:**
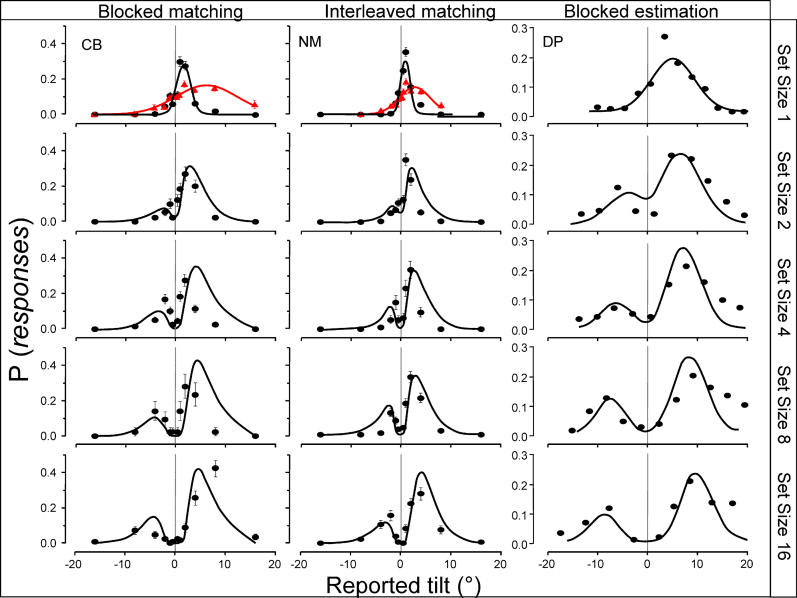
Response Distributions for Near-Threshold Stimuli for Three Observers Three observers' response distributions are shown: CB (left graphs), author NM (middle graphs), and DP (right graphs). For CB and DP, the trials were blocked for set size within each session; for NM, they were randomly interleaved in each session (this had virtually no effect on results). The magnitude matching involved choosing from 12 target tilts; magnitude estimation involved rotating a continuous probe. Each row of plots refers to a particular set size. Each graph plots the proportion of responses to each response probe, collapsing clockwise with counterclockwise stimuli so correct responses become positive and incorrect responses become negative (binning the continuous-probe responses into one-octave logarithmically spaced bins). The positive portions of each distribution are about three-quarters of the total area, reflecting the 67%–83% definition of near-threshold performance. Red triangles in the set size 1 condition show results for orientation-perturbed stimuli, and black circles show normal unperturbed presentations. The error bars show an estimate of standard error of the mean computed by a bootstrap [30] procedure with 1,000 iterations. In the data of the graph at right, we estimated the standard error for both the reported tilt and the bin peak estimate, but they are both smaller than the data points. Note that in these graphs we have rescaled the ordinate as observer DP had lower orientation sensitivity, which caused widening and shortening of the distributions, but showed no difference in the their trend. All curves were tested for bimodality in the following way. The largest positive and negative responses were selected as potential peaks. If any data points between them were significantly lower than both these peaks (bootstrap*t* test,*p* < 0.01) then the distribution was classified as bimodal. All the curves of set size 1, except for CB no-noise, were classified as unimodal. All the curves of larger set size, except NM set size 2, were classified bimodal. The smooth lines show the results of simulation of the signed max model described in the text. It provides good fits to the data, both in predicting uni- and bimodality, and in predicting the separation of the peaks.

During more informal sessions (not recorded) the authors ran the experiment with a colleague providing feedback. It was quite clear that on many trials where the perception was of strong rotation, the target was in fact weakly rotated in the other direction. Naive observers often reported spontaneously that a stimulus seemed very tilted in the condition with a large set size. On debriefing, they reported that the tilt was “real” in all conditions, not qualitatively different as set size varied.

The smooth curves ofFigure 3 show the predictions of the signed max model [9] described in the introduction andFigure 2. The basic assumption of the model is that each stimulus (target and distractor) is monitored by at least two independent noisy detectors (one tuned to clockwise, and another to counterclockwise tilt), and the decision of target tilt will be based on the strongest absolute response from all detectors. The intuition behind the theoretical predictions shown inFigure 2 is that when more detectors are co-opted, the maximum of their (noise-perturbed) responses will necessarily be larger, both in the case on error trials (resulting from a max response given by the oppositely tuned detector) and correct trials (resulting from a max response given by the correctly tuned detector). In the simulation, the output of these detectors is assumed to depend on the physical orientation of the stimulus perturbated by Gaussian noise of standard deviation equal to threshold at set size 1. The orientation of the stimuli was set to 0 for the distractors, while for the target it depended on the actual tilt used to draw the response distributions of each subject. The model chooses the largest absolute tilt away from vertical, conserving its sign. Many such trials (10,000) were simulated to produce the pdfs shown by the continuous lines ofFigure 3. For set size 1, the resultant pdf is obviously the Gaussian perturbation of the single target stimulus. As distractors are introduced, the model searches for the output of largest magnitude, regardless of sign, so the Gaussian perturbation often leads to a distractor having larger magnitude. As a consequence, the “winning” signal will become increasingly larger, so the peaks in the distribution separate further. The data ofFigure 3 fall very close to the predictions of the signed max model. For set sizes greater than 1, the data of all subjects are bimodal, and the two peaks separate with increasing set size.

To bring out better the quantitative predictions of the model,Figure 4 shows how predicted and measured central tendency estimates of perceived tilt vary with set size.Figure 4A plots the means of perceived tilt on error trials (averaged across subjects), showing that these means increase systematically with set size, and that the increase is well predicted by the signed max model.Figure 4B plots the average difference between the modes of the response distributions (like those ofFigure 3), again showing a smooth increase with set size in both data and predictions. Note that the effects are quite large. Average perceived tilt increases by a factor of about four, and intermodal distance by even more.

**Figure 4:**
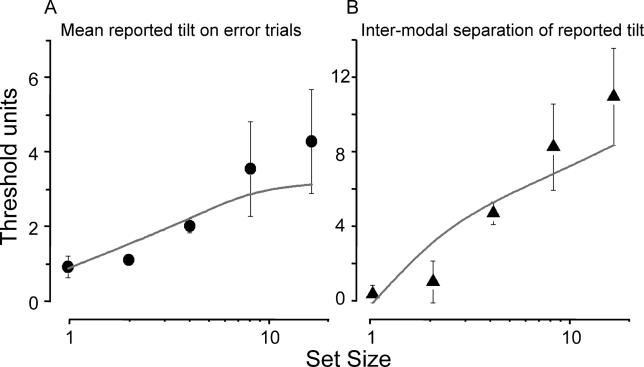
Two Estimates of How Perceived Tilt Varies with Set Size (A) Mean perceived tilt of all erroneous trials in near-threshold conditions, averaged across observers (*n* = 5 at set sizes 1 and 16;*n* = 4 otherwise). The error bars show the standard error of the mean between observers. (B) Distance between the modes of the response distributions (like those ofFigure 3) as a function of set size, averaged across the same observers as shown in (A). The curves of each observer at each set size were first tested for bimodality (see legend toFigure 3). If judged unimodal, the separation was considered zero; otherwise, the distance between the positive and negative peaks was measured and normalized by the individual threshold angle at set size 1. In both plots, the smooth curves show the predictions of simulation of the signed max model, assuming first-stage Gaussian noise of unit standard deviation. The data follow the predictions reasonably well.

The data shown so far refer to near-threshold stimuli: d′ ≅ 1.Figure 5 reports averaged results for near-threshold data for four subjects (those ofFigure 3 plus two not shown), as well as data for subthreshold stimuli. The data for suprathreshold stimuli are not shown as they do not contain a substantial number of errors trials; however, the few errors do follow the predicted pattern. The average results for threshold stimuli (Figure 5B and5D) are similar to the individual results shown inFigure 3: unimodal for set size 1, but clearly bimodal at set size 16. The results for subthreshold stimuli (Figure 5A and5C) are even more interesting. Again both data and predictions (simulations based on average normalized parameters) are Gaussian at set size 1 and bimodal at set size 16, but now the distributions are centered near zero (as the actual stimulus tilt was near zero). The two peaks in the set size 16 condition, corresponding to correct and erroneous judgments, are now nearly equal. Even when no discernable stimulus was physically present, large illusory tilts were perceived, as predicted by the signed max model. We have also looked at response distributions for fixed angle sizes (not shown), which show a very similar trend.

**Figure 5:**
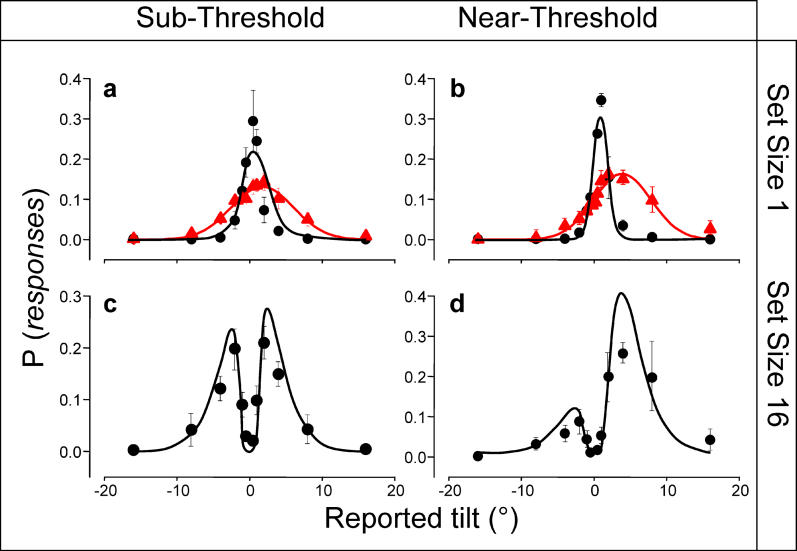
Average Response Distributions for Subthreshold and Near-Threshold Stimuli Distributions are shown at set sizes 1 and 16 (top and bottom row, respectively) for subthreshold (left column) and near-threshold (right column) stimuli. Axes and symbols follow the same convention as inFigure 3. Data show the average of all four subjects, with error bars referring to the standard error of their individual means. At set size 1 the average response distributions are clearly Gaussian-like for both stimulus levels (no curves significantly bimodal), agreeing well with predictions. At set size 16, both distributions are clearly (and significantly) bimodal, again agreeing with predictions.

### Partial Cueing

The experiments reported show that under conditions of visual clutter human observers tend to perceive stimuli to be more strongly tilted on error trials than they do when the stimuli are presented in isolation. One possible confound in interpreting these results is that they could arise directly from low-level sensory interactions between adjacent stimuli akin to “crowding,” caused by the close proximity of stimuli when set size increases. To exclude this possibility, we repeated the crucial conditions using the technique of “partial cueing” [17,18]. Here all displays comprise 16 elements, but either one or all of them were precued (with a high-contrast annulus flashed immediately prior to stimulus onset), as shown in the inserts ofFigure 6. The results of this experiment (Figure 6) are clearly similar to those obtained with the other condition, and close to the theoretical prediction. When only one element was precued, the distribution was unmistakably unimodal, even though the physical arrangement was identical to the set size 16 condition. This excludes the possibility that the bimodality observed in the main experiment arose from orientation interactions between target and distractors, such as orientation contrast or other effects. It shows clearly that the crucial factor producing the bimodality in the response is the number of items attended to, not the number of items per se in the display.

**Figure 6:**
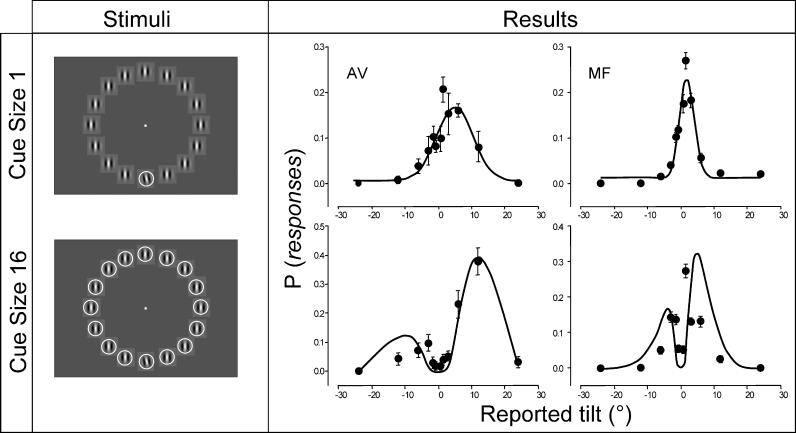
Stimuli and Response Distributions of the Partial Cueing Experiment The left panels show examples of the stimuli used. In the cue size 1 condition (top left panel), 16 elements were displayed and a 2-pixels-thick outlining circle of 1.5° diameter precued (100% valid) the target location, which was randomly set trial by trial. In the cue size 16 conditions, all the patch stimuli were precued. The other four panels show the response distributions for target tilts around threshold for two naive observers, AV (middle panels) and MF (left panels), for the two cueing conditions. The circles represent the proportion of reported responses for each response probe, while the error bars show an estimate of standard error of the means computed by a bootstrap [30] procedure with 1,000 iterations. Although the display has drastically changed, the pattern of results is strictly consistent with that reported inFigures 3 and5, suggesting that the main effect of this study is not due to sensory interactions, or “crowding,” among abutting stimuli.

### Confidence Ratings

For the main experiments of this study we adopted the technique of magnitude matching, which provides metric data that can be modeled parametrically, and which is stable across subjects and over time. Perceived magnitude is known to relate directly to observer confidence [1,2], so these results suggest that confidence also increases with set size. But to be certain, we also measured observer confidence directly with a rating technique: instead of indicating the apparent tilt of the target, observers rated their confidence by clicking an icon of increasing size (seeFigure 1 andMaterials and Methods). The average ratings of four observers for error trials for near-threshold stimuli are shown inFigure 7A. At set size 1, 60% of responses were made with the lowest permissible confidence. At set size 16, only 30% of responses were made with low confidence, with most responses distributed over the higher confidence levels. Average confidence was significantly higher at set size 16 (Figure 7B).

**Figure 7:**
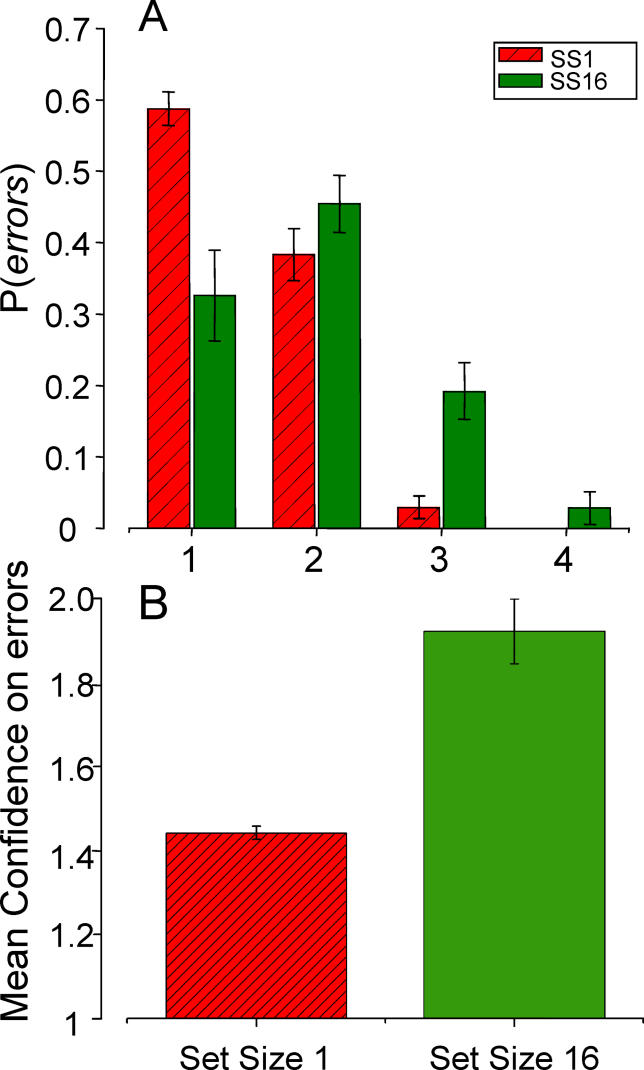
Confidence Ratings for Error Trials at Near-Threshold Tilts The top histogram plots the proportion of responses of error trials at each confidence level, averaged across four observers (all naive of the goals of the experiment). The green bars show responses for set size 16, and the red patterned bars show responses for set size 1, with bars showing ± 1 SEM. In the lowest confidence level, the proportion of errors is higher at set size 1 than at set size 16, while at the three higher confidence levels, the reverse holds. The differences at confidence levels 1 and 3 were statistically significant (Student*t* test,*p* < 0.01). The difference was insignificant at confidence level 2 (where the proportions were similar), and also at level 4 (by binomial test), as there were only five responses in this bin (observers tended to shy away from the response extremes). The bottom bar graphs plot the mean confidence averaged across the same four observers in the set size 1 (patterned red bar) and set size 16 (green bar) condition. The error bars show ± 1 SEM, revealing that our subjects were more confident about their erroneous responses in with a cluttered display than in a single stimulus (Student*t* test,*p* < 0.001).

## Discussion

In this study we tested and substantiated a direct prediction of an SDT-based model that, in a cluttered environment, erroneously perceived stimuli should be seen at higher signal strength than when the target is presented in isolation. The main evidence was the clearly bimodal distributions of reported tilts assessed by magnitude matching and magnitude estimation, where both correct and erroneous responses were signaled with high strength. This suggests that the measured distributions reflect the probable distributions of the internal representation of orientation on which observers base their perceptual decisions in this psychophysical task. The technique of combining magnitude matching with two-alternative forced-choice discrimination provides a far richer data set than the more standard binary response in that it probes the internal probability distributions on which observers base their decisions. There are no strong or unreasonable assumptions inherent in the technique, which may help to yield a clearer idea of the neural representations on which observers base their response.

The data were robust over a wide range of conditions and response techniques. The bimodal distributions occurred both when observers chose from an array of fixed tilts, and when they were free to rotate a dial to any angle. Confidence ratings, while less precise, followed the same general trend. The distributions were bimodal both for tilts near threshold and for subthreshold tilts. Replotting the data at constant tilt angle (rather than relative to threshold) also produced bimodal distributions. Furthermore, the use of partial cueing excluded the possibility that the effect was due to local interactions of neighboring elements, as 16 elements were present in all conditions of this experiment, but no bimodality emerges when only one is cued as the relevant stimulus. These data exclude the possibility that the high-confidence and high-magnitude errors are a consequence of orientation contrast or even “feature migration” [19] between neighboring elements.

The data distributions are well modeled by the signed max model of visual search. This model has been shown to be as good as the ideal observer in similar tasks [20], suggesting that it may have general applicability. The main feature of the model is that it searches for the maximally tilted response among an array of noisy first-stage detectors. The more elements present, the greater (on average) will be the tilt of the most-tilted output, so on both correct and incorrect trials the largest tilt has high magnitude. This occurs even for subthreshold stimuli, where performance approaches chance: most reports tend to be of large angles, whether right or wrong. The results are not simply due to increased task difficulty for large set sizes: randomly perturbing a single target increased thresholds by factors of 3–4, making them compatible with the noise free measurements for set size 16, but the magnitude-estimation distributions remained unimodal.

We chose to model the data with the signed max model, as it has proven to be successful in other situations [9,12]. In this study the model performed very well indeed, not merely predicting the general form of the data, but quantitatively predicting the height and separation of the distribution peaks. Obviously the success of this model does not exclude the possibility of other plausible models, but some models can be excluded outright. For example, the linear summation model favored by many [14,21] could never produce bimodality given the central limit theorem (the sum of many independent random samples tends to Gaussian). Nor is it consistent with high-threshold theories of visual search that assume that errors are due to noisiness at the decision stage (see [7] for review). If this were true, the error functions should be of similar form for all set sizes. It is difficult to see how these classes of models could simultaneously predict both unimodal functions set size 1 (even in the presence of large amounts of noise) and bimodal thresholds for large set size. For the response to be bimodal (with peak separation increasing with set size) the noise must be associated with each stimulus, and must be combined nonlinearly. The nonlinearity in the signed max model is the “maximum” operation that seems to be implemented in the visual system (e.g., [22]), but other physiologically plausible expansive nonlinearities, such as summing after squaring [23,24], will also cause bimodality. The present data does not show a distinction between the various possible underlying nonlinearities, but they do show that some such accelerating nonlinearity is essential.

Whatever the mechanism by which the noisy outputs are combined to produce the bimodal functions, it is clear that the functions must result from bottom-up nonlinear combinations of noisy detectors. One implication of these results is that neural perturbations or “noise” not only lead to perceptual decision errors but, unlike some other noise sources such as photon and photoreceptor noise [25], can be perceived directly and indistinguishably from real orientation signals. The noise in the model is not a theoretical construct: it affects perception directly as a form of an illusion, or self-generated visual perception. This result sits well with a recent fMRI study showing that near-threshold neural signals correlate well with observer response (even when wrong), rather than with physical signal strength [26], and also with older evoked potential studies showing that decisions yielding false alarms are associated with strong auditory neural activity, indistinguishable from real auditory signals [27].

The results imply that visual clutter not only increases errors and raises response thresholds, but also increases the confidence with which observers make decisions. Magnitude matching is an indirect (but stable) way of tapping observer confidence: if unsure of the response, observers press the least-tilted icon available, ± 0.5° (zero was not an option), or set the icon tilt to some small angle (including signed zero). In conditions of low set size, even when noise-perturbed, this is what observers did. That they instead chose icons tilted up to 16°, even in conditions where the target was near vertical or tilted on the other way by the same amount, suggests that they clearly misperceived a highly tilted stimulus that was not there, and did so with high confidence. Although it is intuitively obvious that observer confidence should correlate with perceived signal strength, we also measured confidence more directly by a rating technique. Even though rating scales are inherently less stable than matching tasks and produce nonmetric data, the results with this technique are broadly consistent with the matching results. For the isolated target the confidence ratings tended to be low, while for targets intermixed with distractors the ratings were significantly higher.

These results have practical implications for perceptual decisions in everyday life in that they predict an increase in high-confidence errors when decisions are made in cluttered environments. For example, soccer referees are frequently required to decide rapidly whether a player is “offside” if the ball is passed to him when there are no defenders (besides the goalkeeper) between him and the goal. This study predicts that when there are several candidate defenders that could place the forward onside, the decision will not only be more error-prone, but the confidence with which referees call their (often erroneous) decisions will be higher. Many other simple perceptual judgments in cluttered conditions, such as driving through multiway junctions, could be affected by similar processes, leading to high-confidence errors.

Although this study is limited to simple perceptual decisions about a single stimulus attribute, the same principles could be extended to much more complex decisions involving computations and memory. If the decision-maker has to monitor many events and choose on the basis of magnitude along some dimension, and if each event is perturbed by independent noise, there will be a high probability that the decision, whether right or wrong, will be made with high confidence. High-confidence errors can have major consequences, as American presidential candidate John Kerry mentioned in the first 2004 debate: “It's one thing to be certain, but you can be certain, and you can be wrong. Certainty sometimes can get you into trouble.”

## Materials and Methods

Ten observers participated in these experiments, all aged between 20 and 35 years old, with normal or corrected-to-normal acuity. Two observers were authors (SB and NM); the others were psychology students at the University of Florence, naive to the goals of the study. Author NM (a senior student) was naive to the aims of the experiment when his data were collected. Four subjects (SB, NM, CB, and NF) participated in the magnitude-matching experiment, and the other three (FF, RA, and LM) plus NF (who did both experiments) participated in the confidence-rating experiment while the other two (AV and MF) participated in the partial cueing experiment, which was performed with the magnitude-matching technique. Observer DP participated in the continuous probe experiment.

Stimuli were generated in MATLAB, using Psychophysics Toolbox extensions for Windows [28,29] and presented by PC on a 17” Sony CRT monitor at a 75-Hz refresh rate. Stimuli were Gabor patches (2 c/deg sinusoidal gratings of 50% contrast and 28 cd/m^2^ mean luminance, windowed within a circular Gaussian aperture of 0.5° space constant), arranged at equispaced positions around a notional circle of 5° eccentricity. A stimulus set comprised one to 16 elements, all vertical except for the target, which was tilted clockwise or counterclockwise by 0.5° to 16° (12 possible angles in one-octave steps). In the noise-perturbed condition, the tilt of the target was a sample from a Gaussian distribution with a mean equal to the samples of the noiseless condition and a standard deviation equal to 4°.

Location and orientation of target were randomly assigned on a trial-by-trial basis. Two observers, CB and SB, collected data for different set sizes in different blocks. For the other two observers of the magnitude-matching experiment, NM and NF, the set size was completely randomized within a block of trials without changing the nature of the results. In the confidence rating and the partial cueing experiments, set size was blocked. All sessions comprised 60 trials. The overall number of trials ranged from 400 to 1,200 depending on the stability of the results and the particular condition.

The stimuli were displayed for 100 ms, followed by a mean gray page for 200 ms, and then the response page (Figure 1). In the partial cueing experiment, a circle of 70% contrast surrounded either the target (in the cue size 1 condition) or all the elements (in the cue size 16 condition) 40 ms before trial onset and remained on the screen throughout the stimulus presentation (see insert toFigure 6). Then, a response page appeared. In the discrete magnitude-matching task, subjects were presented with 12 response probes whose orientations corresponded to the stimulus set from which the target was sampled. They responded by mouse-clicking on the one that best matched the perceived target at each trial. In the continuous magnitude estimation task, a response probe resembling the target (but twice as large) appeared and could be rotated through ± 32° by lateral motion of the mouse. In the confidence-rating task, subjects were presented with eight variable-sized icons, all tilted by ± 45°. Observers were all given standardized instructions: “The display contains a number of oriented elements, one of which, the target, is tilted clockwise or counterclockwise off-vertical of various amounts [visual examples provided], while all the others are vertical. The task is to identify the direction of perceived tilt of the target element, which will be displayed in a random location around fixation. [Indicate your response by clicking on one of the clockwise or counterclockwise tilted icons]. [Indicate your response by rotating with the mouse the stimulus until it matches that of the tilted target]. [When responding indicate also the confidence with which you make your decision by clicking on the appropriate sized icon: if you are very sure of your decision, click the largest icon of appropriate tilt; if somewhat unsure click the second-largest icon; if quite unsure, click the second-smallest icon; if very unsure, click the smallest icon.] Try to distribute your responses over the whole response scale.” Subjects were given a practice session to acquaint themselves with the range. Immediately after the response a blank page of 400 ms was displayed and the following trial started automatically (seeFigure 1). No feedback of any kind was given.

## Abbreviations

pdf: probability density function
SDT: signal detection theory
